# A Document of History

**Published:** 1918-07-20

**Authors:** 


### THE BRITISH RED CROSS IN FRANCE.

### A Document of History.

It only needed the recommencement of the German offensive to remind the most thoughtless of us, after the lull in the West, that the celebration of France's Day on July 12 was a national occasion to which in one sense even Independence Day was a secondary event. If France's Day had not been celebrated in this country in previous years, Independence Day could never have been honoured with the same force. And that the meaning of France's Day may be appreciated and preserved, Messrs. Hodder and Stoughton have published a comprehensive survey of British Red Cross activity in France, from the eloquent pen of Mr. Laurence Binyon. "For Dauntless France," as the volume is called, is the official record of the work done by Britain for the French wounded. As M. Cambon, the French Ambassador, remarks in a graceful preface, "the author, who has passed his life in the studious admiration of the Beautiful, has not changed his subject when he here turns to describe so sincerely that which is most beautiful in human life, namely devotion and succour." This tribute to Mr. Binyon is the more graceful in that to set an author of repute to write an official record of events which are too close to be viewed as history, and too scattered to be completely grasped, is to set him a very stiff task. The artist as a writer recoils from the mass of material, the want of perspective, the necessity for introducing so many heterogeneous facts; and yet no record can be official without them. That Mr. Binyon has presented his material with as much "form" as he has is to his credit. His work reads, and the desire to write well is apparent in every chapter.

This acknowledgment is due if we are to appreciate not merely what the presentment of our countrymen's efforts has meant, but even if we are to grasp the extent of the effort with its innumerable ramifications.

Mr. Binyon begins by describing the confused scene after the war started: the volume of sympathy only matched by the absence of organisation; the difficulty of co-ordinating effort; the disparity between the confused crowd of waiting helpers and the need which could not wait upon time. Luckily, England was better prepared than her ally in one direction. The number of trained nurses in this country was great. In France, where nursing had been mainly in the hands of the religious sisterhoods, the expulsion of the nuns had left a big gap to be filled. Since, too, voluntary helpers were not welcomed at first by our own military authorities, there was an opening for trained voluntary units in France. Women suffragists, even Ulster organisations, turned their resources to use or sent their medical corps already equipped to the aid of the French soldiers. There indeed existed the three Red Cross Societies of France: La Société de Secours aux Blessés Militaires, the Association des Dames Françaises, and the Union des Femmes de France, of which the last, luckily, possessed a committee in London. In the early days voluntary units found France easy to enter: the crisis came first, the regulations afterwards, and it was not till 1915 that a necessary and desirable certificate was issued to all recognised workers by the Anglo-French Hospitals Committee, under the Joint Societies here. Thus an easy way to abuse the Red Cross was in time prevented, and meantime the three French Societies already named had been, of course, most rigorously at work. If an unforeseen shortage of nurses had been produced by recent French legislation, the three French Societies were, better than our own, prepared for war. The impulse of 1870 had not been spent, and every town in France had its own branch which could place, it was believed, a sufficiency of beds at the disposal of the French Service de Santé. War is always full of surprises, however, and how much suffering lies silent behind the simple statement that the event proved on a larger scale than had been foreseen! What France lacked in number of trained nurses she possessed in workers like our own V.A.D.s, who, unlike ours, had received several months' training, and were ready for the call.

A Front nowadays is as long as a continent. Each sector has its own army, its own needs, its own geography, its own weather. To meet the different problems so produced Red Cross work is equally varied. Mr. Binyon takes us through the London office, and the Flanders mud, to the snowy slopes of the Vosges, behind Verdun, on motor ambulances and a river barge, up hillsides in a Red Cross side-car, through heat and cold into theatres behind the lines where a concert is given by some Red Cross staff to the children of a French village, on the crowded roads seething with traffic behind an active part of the Front, among the hospitals that lie behind, and on the trains which bring the wounded into safety. It is a picture of incessant, seemingly confused activity, an ordered disorder as of waves breaking upon a beach. Its details make it real and hold the reader. Every phase has its own pages, every picture in its degree is complete. We follow this or that section hither and thither as it moves with the division to which it is attached. We remain with another for long periods on the same sector of the line. We learn, too, sometimes of the French native African regiments, of their prisoners in Germany, of the prevalence of trench feet among the Moroccans, small details which flash the scale of the war before our eyes.

In places where "there is nothing to report" men are always being wounded, and no Front is too "quiet" to be casualty-free. Since the English drivers rank as privates in the French Army they receive the regular pay of five sous a day and army rations which the men's funds supplement. At times when roads in Flanders were impassable for

**The British Red Cross in France—(*continued*).**

cars "young German prisoners were requisitioned to help to carry the wounded over the chaos of mud and water, and, famished though they were, did the work well." Among the doings of the many convoys described is the varied activity of "Fanny," composed of Englishwomen and officially known as the First Aid Nursing Yeomanry Corps.

Special chapters are devoted to the hospital supply depôts and the work of our countrymen in or for the 4,307 military hospitals of France. The hospital story begins with the conversion of the Queen Victoria Memorial Hospital at Nice to military use on August 3, 1914. The stunning fall of Namur—so unthinkable that "no plans for an orderly retreat had been made "—found and left the English nurses with their wounded. Hospital after hospital is described in the order of its foundation, and no one will complain of dullness in the long and varied list. The life of the orderly, the life of a canteen worker, what relief work means behind the lines which the sway of the fighting has left desolate, form separate chapters, perhaps the most interesting of the book. But some who perhaps have a regard for Mr. Binyon as a writer and remember, say, his study of Chinese art, may expect to see him most untrammelled in the concluding section of the volume, where he groups under the title of "Impressions" a series of descriptions of what he has seen himself in different parts of France, where an old Frenchwoman's story, a canteen in a forest, a visit to Gerbéviller (the town which was scientifically sacked), has fixed itself in his memory. These impressions speak for themselves and relieve the ordered story of the detailed convoys, hospitals, and ambulances wherein Englishmen, often volunteers, have worked for France in a fine spirit of patriotism. The statistical index, the full list of British subjects engaged on Red Cross work in France, remind us in conclusion that we are reading an official story, which will be one of the "documents" of the war, compiled by one who has seen those of whom he writes in the thick of their work, and has brought to its description considerable technical ability. It is a magnificent record of personal service of which Englishmen may be proud, since it is the hospital spirit on active service which animates the immense field of Red Cross activity; and of this the part played by Englishmen is necessarily the only part told. May the French Red Cross Organisations find an equally capable historian!

